# Evaluation of *in vitro* and *in vivo* antibiotic efficacy against a novel bioluminescent *Shigella flexneri*

**DOI:** 10.1038/s41598-019-49729-2

**Published:** 2019-09-19

**Authors:** Molly C. McCloskey, Shareef Shaheen, Lesley Rabago, Matthew A. Hulverson, Ryan Choi, Lynn K. Barrett, Samuel L. M. Arnold

**Affiliations:** Department of Medicine, Division of Allergy and Infectious Diseases, and the Center for Emerging and Re-emerging Infectious Diseases (CERID), University of Washington, Seattle, WA 98109 United States

**Keywords:** Drug screening, Intestinal diseases, Preclinical research

## Abstract

*Shigella* spp., the bacteria responsible for shigellosis, are one of the leading causes of diarrheal morbidity and mortality amongst children. There is a pressing need for the development of novel therapeutics, as resistance of *Shigella* to many currently used antibiotics is rapidly emerging. This paper describes the development of robust *in vitro* and *in vivo* tools to study antibiotic efficacy against *Shigella flexneri*. A novel bioluminescent *S. flexneri* strain (*S. flexneri* lux1) was generated, which can be used in a mammalian epithelial cell co-culture assay to evaluate antibiotic intracellular and extracellular efficacy. In addition, the *S. flexneri* lux1 strain was used with an intraperitoneal (IP) murine model of shigellosis to test the efficacy of ciprofloxacin and ampicillin. Both antibiotics significantly reduced the observed radiance from the gastrointestinal tissue of infected mice compared to vehicle control. Furthermore, plated gastrointestinal tissue homogenate confirmed antibiotic treatment significantly reduced the *S. flexneri* infection. However, in contrast to the results generated with tissue homogenate, the radiance data was not able to distinguish between the efficacy of ampicillin and ciprofloxacin. Compared to traditional methods, these models can be utilized for efficient screening of novel antibiotics aiding in the discovery of new treatments against shigellosis.

## Introduction

In resource limited areas such as sub-Saharan Africa, Asia, and Latin America, shigellosis is a frequent and persistent cause of diarrhea^1,2^. Infection by organisms of the genus *Shigella*, the Gram-negative facultative bacteria responsible for shigellosis, has emerged as one of the leading causes of diarrheal morbidity and mortality in children under the age of five^2,3^, and a recent analysis suggests that as many as 164,000 deaths per year are attributed to the disease^1^. Shigellosis is often characterized by watery, mucoid, or bloody diarrhea and can lead to fever and tenesmus in severe cases^4,5^. There are four serogroups of *Shigella*, including *S. sonnei*, *S. dysenteriae*, *S. boydii*, and *S. flexneri*^6^. While *S. sonnei* is commonly seen in industrialized countries, *S. flexneri* poses a significant risk in developing areas of the world^7^. *S. dysenteriae* is especially dangerous as the bacteria produces Shiga toxins (Stx) and is usually involved in epidemic outbreaks^8^. However, new cases of shigellosis caused by *S. dysenteriae* seem to be largely diminished^9,10^.

Currently, there are no approved human vaccines for shigellosis, and interspecies variation may make it difficult to develop a vaccine that protects against all or most *Shigella* strains. In addition, *Shigella* has demonstrated an ability to rapidly acquire antibiotic resistance, with widespread resistance to many antimicrobial agents used for empirical therapy. Recently, resistance to ciprofloxacin^11,12^, the WHO-recommended antibiotic for treatment of dysentery^13^, as well as resistance to azithromycin^14–16^, was reported. As such, it is of crucial importance to determine new potential treatments for this disease, and antibiotics are still one of the best defenses against shigellosis. Therefore, the aims of this work were to develop improved methods and tools to rapidly test new potential antibiotics for the treatment of shigellosis.

Presently, there is a lack of informative cost effective and high throughput *in vitro* tools to assay antibiotic efficacy against *Shigella* infection. Minimum inhibitory concentration (MIC) assays allow for assessment of *Shigella* susceptibility to antibiotics, but how the MIC values translate to *in vivo* efficacy is often unclear. The efficacy of antibiotics against intracellular bacteria such as *Shigella* is of particular interest. Initially developed in 1964, epithelial cell invasion assays have been instrumental in characterizing the phenotype of virulent *Shigella*^17^. The invasion assays are based on a co-culture system using mammalian cells to mimic the site of *Shigella* invasion *in vivo*. Although there are many well recognized limitations to the invasion assay, these assays still play a crucial role in our basic understanding of *Shigella* invasion, spread, and intracellular replication. Here, to the best of our knowledge, we are the first to use the co-culture invasion assay to characterize the efficacy of antibiotics against *Shigella in vitro*.

While multiple methods of quantification have been established for monitoring *Shigella* levels, these approaches do not provide robust and sensitive methods to quantify viable *Shigella in vitro* and *in vivo*. The traditional technique of plating tissue homogenates or *in vitro* samples provides a sensitive method to quantify the levels of viable *Shigella*, but this method is laborious and not conducive for high throughput analysis of *Shigella* levels. qPCR assays provide a sensitive method that can be adapted to high throughput systems, but the assays cannot distinguish between live and dead bacteria. Green fluorescent protein (GFP)-expressing variants of *S. flexneri* have been developed and used *in vitro* and *in vivo*^18^. However, the use of GFP protein for detection and/or quantitation can be hindered by photobleaching and persistence. Previous work with the enteric pathogen *Salmonella*^19,20^ and enteropathogenic *Escherichia coli*^21^ established bioluminescence as a reliable method to monitor real-time levels of live bacteria *in vitro* and *in vivo*, but there are no published studies that describe the characterization of bioluminescent *Shigella*. Therefore, to improve the current options for *Shigella* detection, a strain of *S. flexneri* was generated that contains a stable bioluminescence-based reporter system based on previous work with *Salmonella enterica*^19^.

Although the lack of an adult mouse model has made studying this disease quite difficult in the past, we further developed a recently published intraperitoneal (IP) infection mouse model of shigellosis^18^. While other groups have used the model for vaccine development^18,22,23^, we utilized the mouse model to evaluate antibiotic efficacy against *Shigella*. This model was used along with the bioluminescent *S. flexneri* lux1 strain to efficiently evaluate antibiotic efficacy of two well characterized drugs, ciprofloxacin and ampicillin.

## Results

### Detection, quantification, and growth kinetics of *S. flexneri* expressing bacterial luciferase

**Figure 1:**
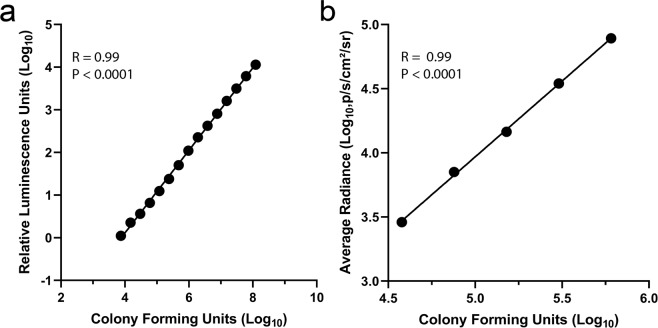
Characterization of *S. flexneri* lux1 calibration curves. The log transformed *S. flexneri* lux1 relative luminescence units (RLU) were plotted as a function of log transformed CFUs. The limit of detection was 7,500 CFUs with the Perkin Elmer Envision system (**a**). The experiment was repeated and analyzed with a Perkin Elmer IVIS Spectrum (**b**). With the IVIS Spectrum, the limit of detection was 47,315 CFUs. Linear regression was used to identify an association between the luminescence and CFUs. Each point represents the average of three technical replicates.

To generate a strain of *S. flexneri* that expresses luciferase, plasmid pBEN276 was transformed into streptomycin-resistant M90T Sm *S. flexneri*. The *luxCDABE* genes were transposed into the bacterial chromosome at the *attTn7* site, and the bacteria was cured of the plasmid. A clone was selected and characterized for further analysis. To initially characterize the association between luminescence and CFUs, a Perkin Elmer Envision was used to quantify the luminescence of *S. flexneri* lux1. The bioluminescence readings from the dilutions of *S. flexneri* lux1 in a 96-well white plate demonstrated a linear relationship to bacteria number (R^2^ = 0.99, *P* < 0.0001) when both values were log transformed. The minimum detectable CFU was 7,500 for this assay (Fig. 1a). To characterize the detection of *S. flexneri* lux1 with the IVIS Spectrum system, log transformed average radiance measurements from *S. flexneri* lux1 were plotted against log transformed bacteria number (CFUs) (Fig. 1b). There was a significant, positive relationship between the observed radiance and the CFUs (R^2^ = 0.99, *P* < 0.0001). However, the minimum detectable CFU was >6-fold higher (47,315 CFU) with the IVIS Spectrum compared to the Perkin Elmer Envision.

**Figure 2:**
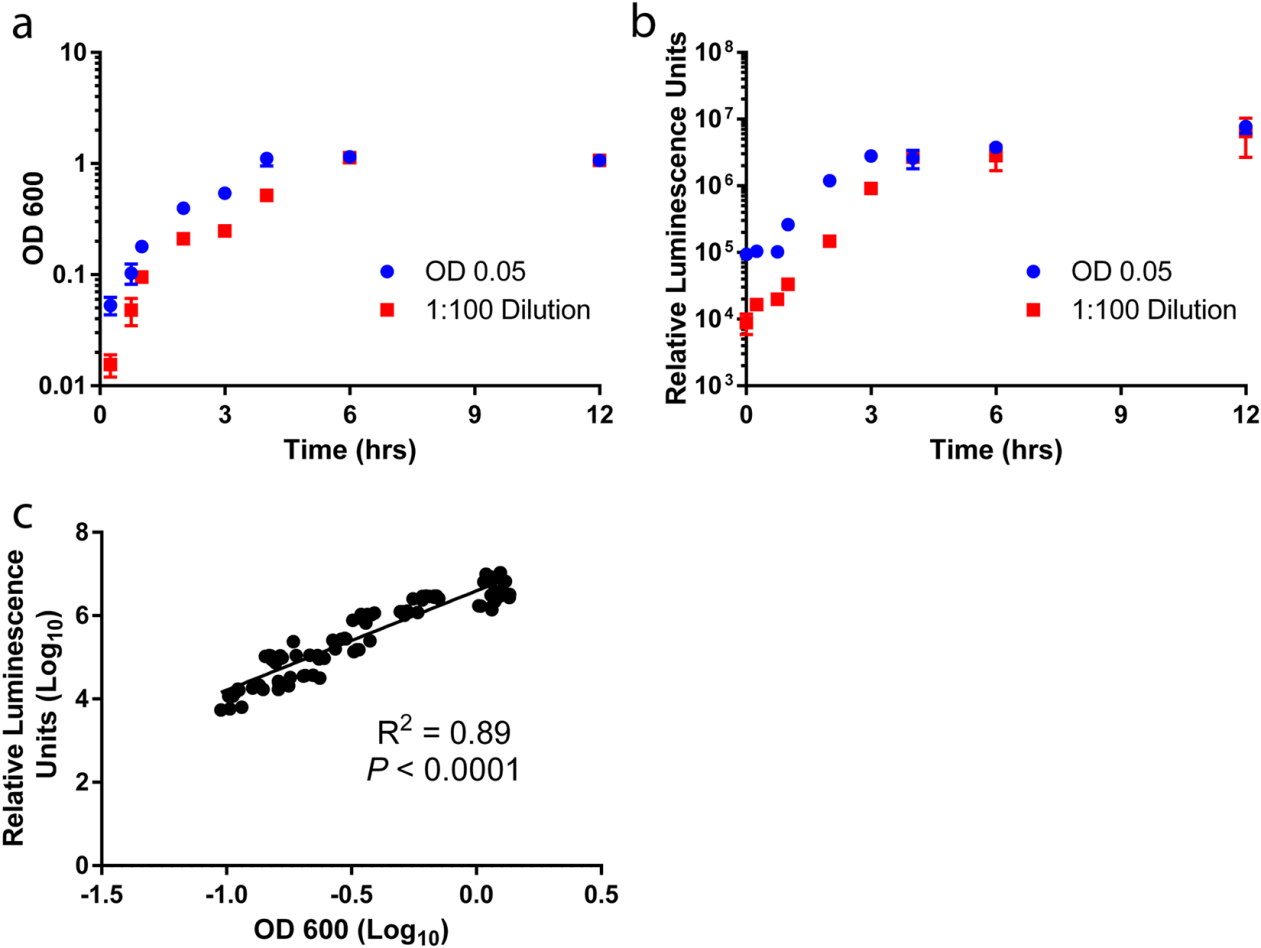
Characterization of *S. flexneri* lux1 growth kinetics. An overnight culture of *S. flexneri* lux1 was diluted 1:100 and to a nominal OD600 of 0.05. The cultures (n = 3 for each dilution) were shaken at 37 °C and aliquots were removed at multiple time points. The OD600 (**a**) and luminescence (**b**) were measured at each time point. Values are means ± SD. A significant positive association (R^2^ = 0.89, *P* < 0.0001) between the luminescence and OD600 was observed when the log transformed relative luminescence units (RLUs) were plotted against the corresponding log transformed OD600 values (**c**). Linear regression was used to identify an association between the luminescence and OD600.

To characterize the growth kinetics of *S. flexneri* lux1, the luminescence of *S. flexneri* lux1 cultures was monitored over 12 hours. An overnight culture of *S. flexneri* lux1 was diluted either 1:100 or to a nominal OD600 of 0.05. With our growth conditions, all overnight cultures of *S. flexneri* lux1 have an OD600 of ~0.8. The diluted cultures were grown for 24 hours with frequent OD600 and bioluminescent readings over the first 12 hours (Fig. 2a,b). According to the OD600 data, the stationary phase was reached by 4 hours when the overnight culture was diluted to an OD600 of 0.05. However, when the overnight culture was diluted 1:100, the stationary phase was reached by 6 hours. In agreement with the OD600 data, the luminescence readings suggested the stationary phase was reached by 4 hours when the overnight culture was diluted to a nominal OD600 of 0.05. However, the luminescence data suggested the 1:100 dilution cultures reached the stationary phase by 4 hours which was 2 hours earlier than observed with the OD600 reading. Overall, for all samples collected from the cultures over 12 hours, there was a significant positive association between the log transformed luminescence and log transformed OD600 values demonstrating that the luminescence is associated with the number of *S. flexneri* (Fig. 2c). For both dilutions, there was no luminescence detected at 24 hours, potentially pointing to a decrease in translation or transcription of *luxCDABE* during stationary phase or that the protein has shorter stability than the biomass of the bacteria (data not shown). Additionally, this loss of luminescence may be due to the natural death phase of the bacteria. Since the *in vitro* antibiotic efficacy experiments with HCT-8 cells were performed in under 5 hours and given that bacteria would be actively replicating in the *in vivo* model, these properties of luciferase activity were considered sufficient to monitor *in vitro* log phase growth as well as *in vivo* infection.

### *In vitro* characterization of antibiotic efficacy against *S. flexneri*

To initially characterize the *in vitro* efficacy of antibiotics, MIC assays were used to test multiple classes of antibiotics. As expected, the parental M90T Sm *S. flexneri* and luciferase-expressing *S. flexneri* had identical sensitivities to the antibiotics tested, demonstrating that insertion of *luxCDABE* did not change the susceptibility of the bacteria to antibiotics (Table 1).

**Table 1:** Antibiotic potency against *S. flexneri* M90T Sm and *S. flexneri* lux1.

Antibiotic	Class	MIC (µg/mL)	
		M90T Sm	lux1
Ampicillin	Penicillin	1	1
Gentamicin	Aminoglycoside	8	8
Streptomycin	Aminoglycoside	>64	>64
Ciprofloxacin	Fluoroquinolone	0.008	0.008
Ceftriaxone	Cephalosporin	0.03	0.03
Cefamandole	Cephalosporin	0.25	0.25
Cefotaxime	Cephalosporin	0.02	0.02
Pivmecillinam	Mecillinam Prodrug	0.5	0.5
Nitrofurantoin	Nitrofuran	8	8
Furazolidone	Nitrofuran	2.5	2.5

**Figure 3:**
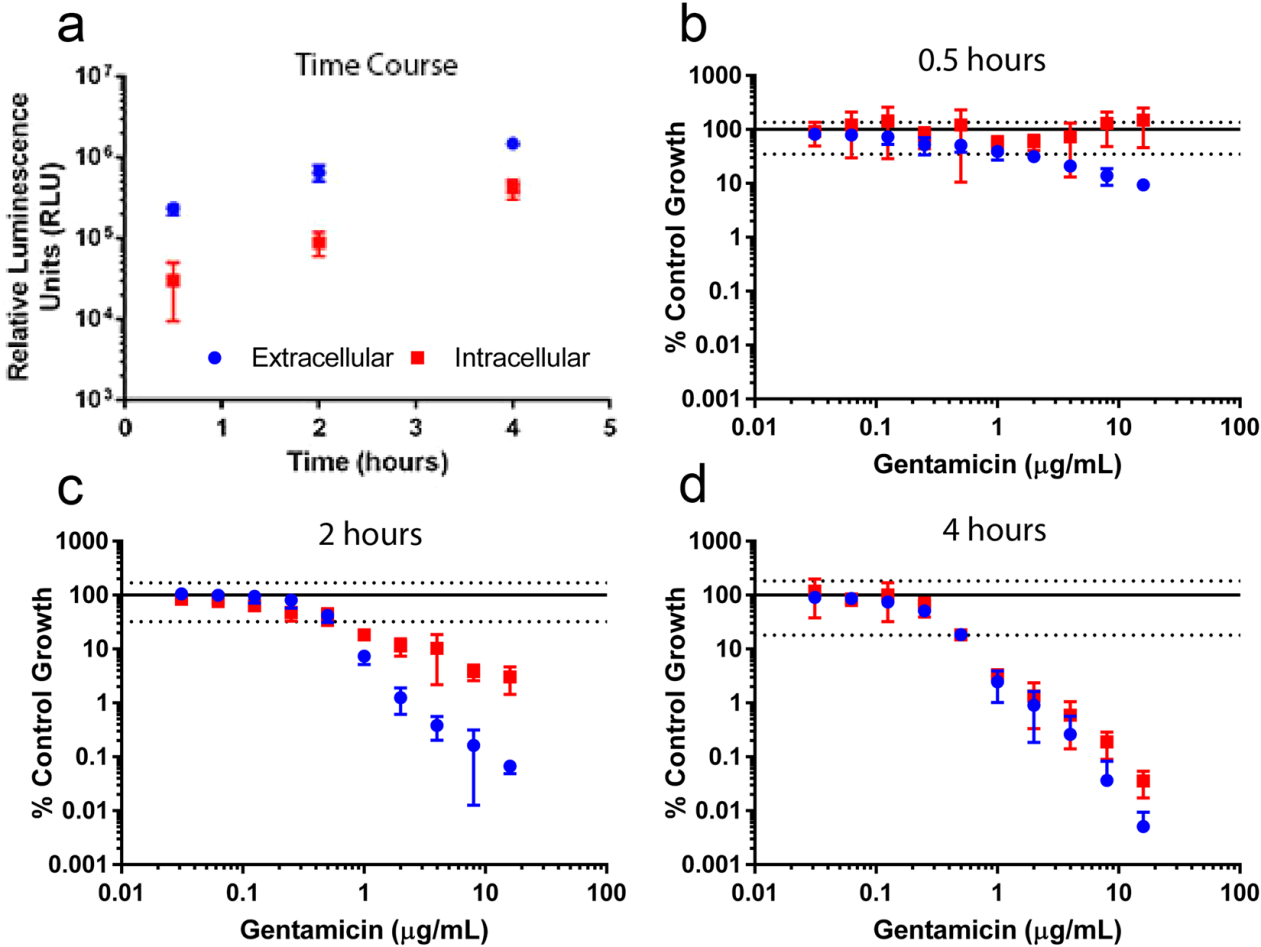
Time course analysis of gentamicin’s effect on extracellular and intracellular *S. flexneri*. A mammalian cell co-culture time course assay with HCT-8 cells and *S. flexneri* lux1 was used to monitor *S. flexneri* intracellular (red square) and extracellular (blue circle) growth and invasion (**a**). The time course assay was repeated with *S. flexneri* lux1 in the presence of gentamicin for 0.5 (**b**), 2 (**c**), or 4 (**d**) hours. The RLU values of the gentamicin-treated wells were normalized to wells that contained vehicle control. Values are means ± SD (n = 8). The solid line and dashed lines represent the normalized mean ± SD of the vehicle control (extracellular samples) at each time point, respectively. The vehicle control relative luminescence unit SD for extracellular and intracellular samples were similar at each time point.

To study antibiotic efficacy against both extracellular and intracellular bacteria, the mammalian cell line HCT-8 was inoculated with *S. flexneri* lux1 and treated with antibiotics. To initially assess invasion of *S. flexneri* lux1 prior to antibiotic evaluation, a time course analysis was performed by adding bacteria to the HCT-8 cells, allowing 30 minutes for bacterial invasion, and then quantifying bacteria in the media and in the cell lysate via luminescence after an additional incubation of 0.5, 2 and 4 hours (Fig. 3a). Extracellular and intracellular bacteria levels were significantly higher at each consecutive time point (*P* < 0.05).

**Figure 4:**
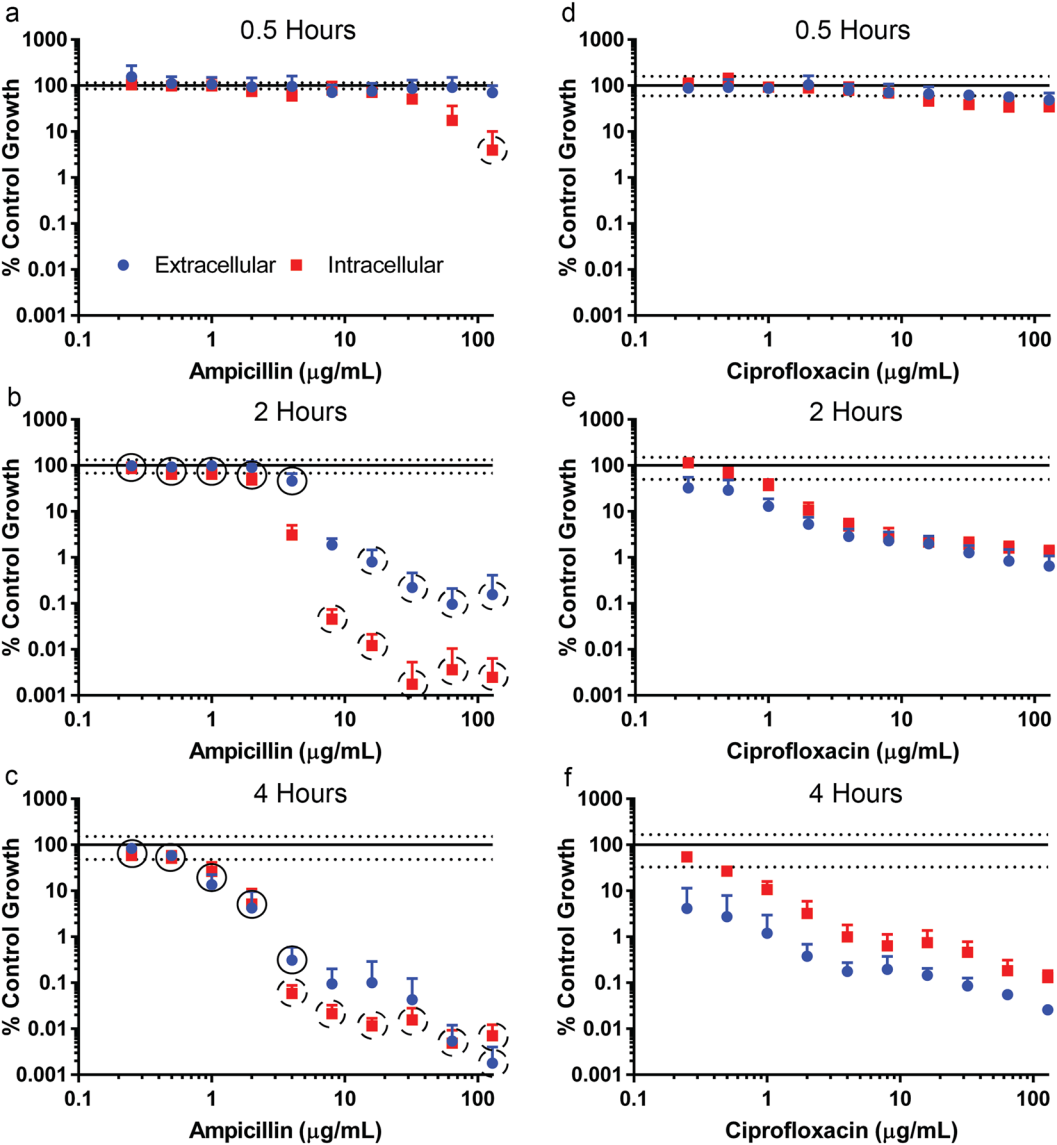
Mammalian cell co-culture assay with *S. flexneri* lux1 in the presence of ampicillin or ciprofloxacin. The luminescence of the intracellular (red square) and extracellular (blue circle) *S. flexneri* was measured after incubation with either ampicillin (**a**–**c**) or ciprofloxacin (**d**–**f**) for 0.5 hours (**a**,**d**), 2 hours (**b**,**e**), and 4 hours (**c**,**f**). The values of the antibiotic treated wells were normalized to wells that contained vehicle control. Values are reported as mean ± SD (n = 8). The solid line and dashed lines represent the normalized mean ± SD of the vehicle control (extracellular samples) at each time point, respectively. The vehicle control relative luminescence unit SD for extracellular and intracellular samples were similar at each time point. A black circle with a solid line represents a significantly larger reduction in luminescence generated by ciprofloxacin compared to ampicillin at the corresponding concentration. In contrast, a black circle with a dashed line represents a significantly larger reduction in luminescence generated by ampicillin compared to ciprofloxacin at the corresponding concentration.

As traditional invasion assays use gentamicin to clear extracellular bacteria, an additional time course assay was performed with the antibiotic plated in serial 2-fold dilutions. The luminescence signals of intracellular and extracellular bacteria were measured at 0.5, 2, and 4 hours after the addition of gentamicin (Fig. 3b–d). Although there was no effect on intracellular bacteria at 0.5 hours, gentamicin concentrations ≥0.125 µg/mL significantly reduced extracellular bacteria compared to antibiotic-free control (*P* < 0.05). At 2 hours, a concentration-dependent reduction was seen in both extracellular and intracellular *S. flexneri* compared to antibiotic-free controls with gentamicin concentrations ≥0.125 µg/mL. In addition, extracellular *S. flexneri* was reduced significantly more than intracellular *Shigella* with gentamicin concentrations ≥1 µg/mL (*P* < 0.001). By 4 hours, intracellular and extracellular *S. flexneri* were both reduced in a comparable manner, and a significant difference (*P* < 0.001) between intracellular and extracellular *S. flexneri* reduction was only observed at 16 µg/mL. To further characterize antibiotic efficacies against *S. flexneri*, ampicillin and ciprofloxacin were tested in the mammalian cell co-culture assay (Fig. 4). Following bacterial invasion, antibiotics were plated in serial 2-fold dilutions and plates were incubated an additional 0.5, 2, and 4 hours. Although both antibiotics affected intracellular and extracellular bacteria, the predominant localization of the antibiotic effect was different. At 0.5 hours, ampicillin had a significantly (*P* < 0.001) greater effect on intracellular bacteria compared to extracellular bacteria at concentrations ≥64 µg/mL (Fig. 4a). The concentration required to observe a significant difference was decreased to concentrations ≥4 µg/mL at 2 and 4 hours (Fig. 4b,c). In contrast to ampicillin, ciprofloxacin did not generate a 10-fold reduction in extracellular or intracellular bacteria at 0.5 hours (Fig. 4d). Furthermore, extracellular levels of bacteria were decreased more than intracellular bacteria at 2 and 4 hours (Fig. 4e,f). However, for ciprofloxacin, extracellular bacteria levels were significantly (*P* < 0.01) less than intracellular bacteria at all concentrations tested in the assay at 4 hours (Fig. 4f). Although the extent of *Shigella* reduction by ciprofloxacin is not as large as ampicillin at higher concentrations, low concentrations of ciprofloxacin has a larger effect on *Shigella* compared to ampicillin. The only tested antibiotic concentration to generate a significant difference (*P* < 0.001) between the two antibiotics in bacteria reduction at 0.5 hours was 128 µg/mL, and this difference was only observed with intracellular bacteria (Fig. 4a,d). However, at 2 hours, there was a significant difference (*P* < 0.0001) between each antibiotic’s reduction of extracellular *S. flexneri* at each tested concentration with the exception of 8 µg/mL (Fig. 4b,e). A similar trend was observed with intracellular bacteria at 2 hours, but the lack of significance was observed at 4 µg/mL instead of 8 µg/mL. Finally, at 4 hours, ciprofloxacin significantly (*P* < 0.0001) generated a larger reduction in extracellular *S. flexneri* compared to ampicillin with concentrations between 0.25–4 µg/mL (Fig. 4c,f). However, this relationship reversed with antibiotic concentrations ≥64 µg/mL. In addition, at 4 hours, ciprofloxacin significantly (*P* < 0.0001) generated a larger reduction in intracellular *S. flexneri* compared to ampicillin with concentrations between 0.25–2 µg/mL. In contrast, ampicillin generated a significantly larger reduction in intracellular *S. flexneri* levels at antibiotic concentrations ≥4 µg/mL compared to ciprofloxacin.

### Development of a murine model of shigellosis for antibiotic efficacy

To complement our *in vitro* studies, a murine model of shigellosis was used for the evaluation of antibiotic efficacy *in vivo*. Initially, IFN-γ KO mice were evaluated as a potential mouse shigellosis model with escalating oral doses of *S. flexneri* lux1. Twenty-four hours after inoculation there was no significant difference in the radiance observed in the gastrointestinal tissue collected from the control mice versus IFN-γ KO mice administered *S. flexneri* lux1 (*P* > 0.05) (Supplemental Fig. 1a). However, there was a small (*P* < 0.01) significant difference in the weight changes between control and infected mice (Supplemental Fig. 1b). In agreement with the radiance data, *S. flexneri* was not detected when gastrointestinal tissue homogenate was plated on agar plates (data not shown).

**Figure 5:**
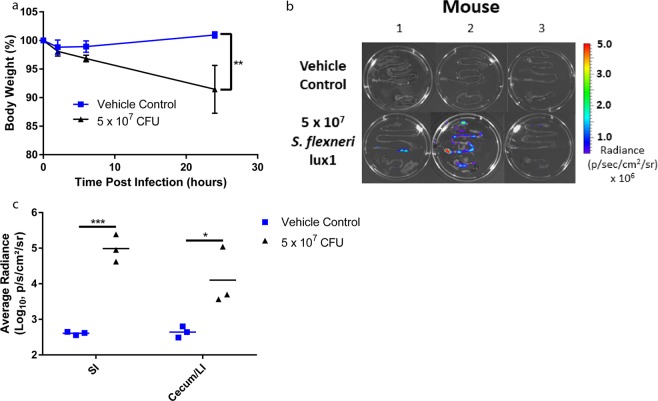
*Shigella* infection in the SI and cecum/LI of adult mice 24 hours after IP infection with *S. flexneri* lux1. Mice were administered vehicle control or 5 × 10^7^ CFU *S. flexneri* lux1 (n = 3 mice/group), and their weights were monitored over a 24-hour period (**a**). To determine if gastrointestinal tissue from infected mice had detectable luminescence at 24 hours post infection, the small and large intestines were collected from mice administered vehicle control or *S. flexneri* lux1 (n = 3 mice/group) and were imaged with an IVIS Envision Spectrum (**b**). There was significantly higher radiance (*P* < 0.05) measured from the gastrointestinal tissue of mice administered *S. flexneri* lux1 compared to mice administered vehicle control. For the control and infected mice, the weights and tissue radiance were compared with a Student’s two tailed t-test. *P < 0.05, **P < 0.01, ***P < 0.001.

Due to the lack of infection with oral administration of *S. flexneri* to IFN-γ KO mice, a previously published intraperitoneal model of shigellosis with B6 mice was modified to evaluate antibiotic efficacy^18^. To identify an optimal dose for IP administration of *S. flexneri* to B6 mice, a dose escalation study was performed with *S. flexneri* lux1. Mice were dosed IP with vehicle control or *S. flexneri* lux1 (5 × 10^7^, 5 × 10^8^, or 1 × 10^9^ CFUs). Of the three dose groups, only mice from the lowest infection level (5 × 10^7^ CFU) survived for 24 hours along with the uninfected controls. In addition, the infected mice lost significantly (*P* < 0.01) more weight compared to the vehicle controls (Fig. 5a). To determine if *S. flexneri* lux1that localized to the gastrointestinal tissue of infected mice could be monitored with an IVIS Spectrum, the experiment was repeated with IP administration of vehicle control or 5 × 10^7^ CFUs *S. flexneri* lux1. 24 hours post infection (p.i.), the gastrointestinal tract was removed from each mouse in the study (n = 3 mice/group). When the gastrointestinal tissue was imaged with IVIS, there was a small amount of background radiance observed in the uninfected mice (Fig. 5b,c). However, the average radiance observed from the small and large intestine of mice administered 5 × 10^7^ CFU *S. flexneri* lux1 was significantly higher (*P* < 0.05) than uninfected mice (Fig. 5c), suggesting *S. flexneri* lux1 could be used to study antibiotic efficacy against *S. flexneri in vivo*.

### *In vivo* screen of antibiotic efficacy against *S. flexneri*

**Figure 6:**
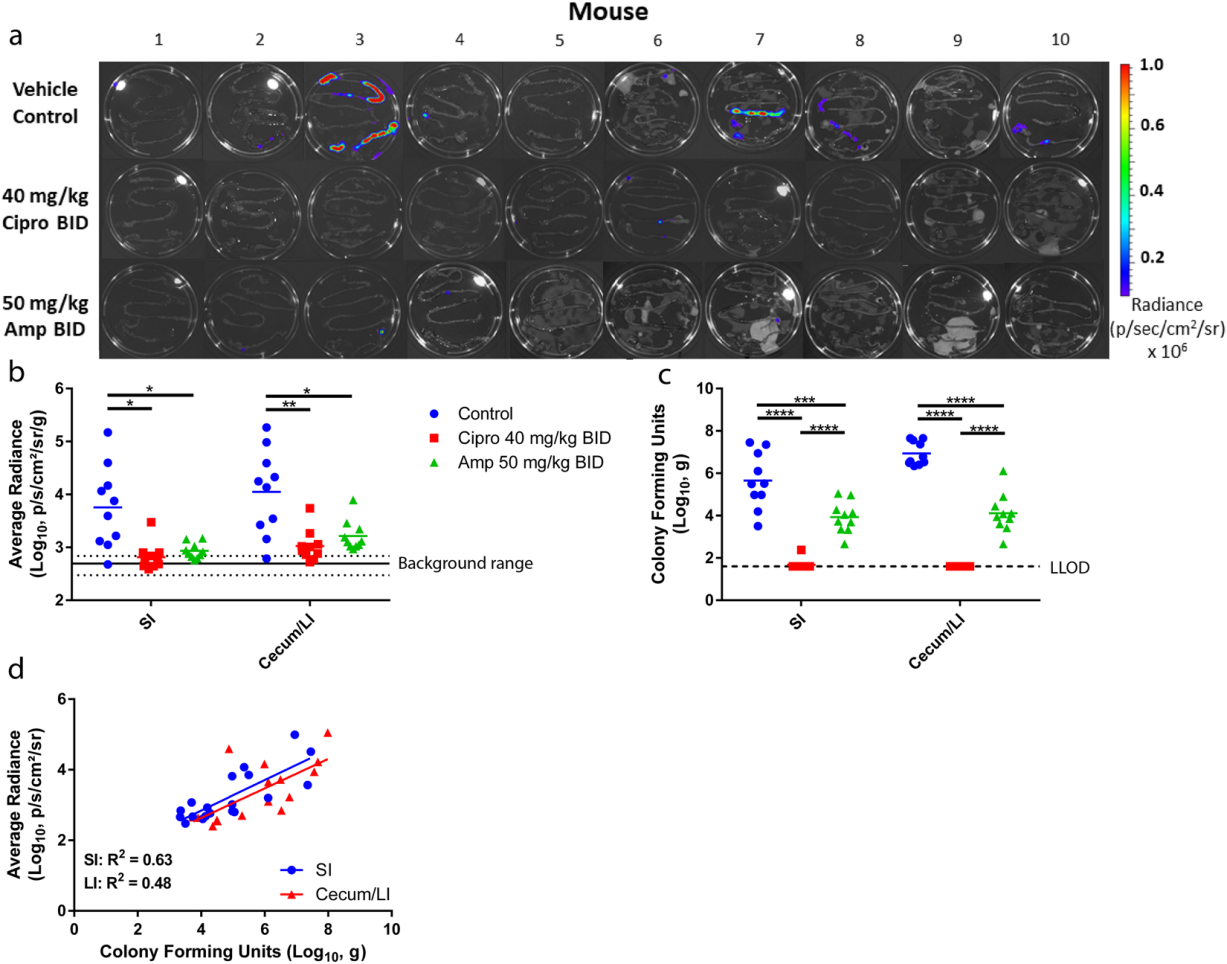
Efficacy of ciprofloxacin and ampicillin in the mouse model of *Shigella* infection. Adult mice were infected IP with *S. flexneri* lux1 (5 × 10^7^ CFU/mouse) and treated orally with vehicle control, ciprofloxacin (40 mg/kg), or ampicillin (50 mg/kg). The gastrointestinal tract was removed from the mice 24 hours post infection, and the level of *S. flexneri* infection was determined by IVIS (**a,b**) and plating tissue homogenate (**c**). The average background radiance is represented with a solid line in panel b and the dashed lines are the SD. For the tissue homogenates, the dashed line is the lower limit of detection (LLOD). For both the IVIS data and the plated tissue homogenate data, an ANOVA analysis with post-hoc comparison identified a significant (*P* < 0.05) reduction in the infection of antibiotic treated mice compared to vehicle treated controls. However, a Student’s two tailed t-test only identified a significant difference between the antibiotic treated groups with data generated from the plated tissue homogenate (*P* < 0.0001). To determine if there was an association between the tissue radiance and the CFUs, the average radiance measurements of the SI (blue circle) and cecum/LI (red triangle) were plotted against their corresponding homogenate CFU (**d**). There was a significant, positive association between the log transformed observed radiance and the log transformed measured CFU (SI, R^2 = ^0.63, *P* < 0.0001, cecum/LI, R^2^ = 0.48, *P* < 0.01). Experiments were performed in duplicate on separate days (total n = 10 mice/per group, with n = 5 mice/group for each duplicate). *P < 0.05, **P < 0.01, ***P < 0.001, ****P < 0.0001.

The efficacy of the antibiotics ciprofloxacin and ampicillin were assessed in the mouse model of shigellosis using *S. flexneri* lux1. Following IP administration of 5 × 10^7^ CFU/mouse, mice were dosed twice via oral gavage (PO) with ciprofloxacin (40 mg/kg) or ampicillin (50 mg/kg). 24 hours p.i., the mice were euthanized, and their gastrointestinal tissue was collected. Within 10 minutes of collection, the gastrointestinal tissue was imaged with the IVIS Spectrum (Fig. 6a). An ANOVA analysis was used to identify a significant reduction in the bacterial counts of antibiotic treated mice compared to vehicle controls (*P* < 0.05). However, a Student’s two-tailed t-test did not identify a significant difference between the antibiotic treatments (*P* > 0.1) (Fig. 6b). Except for one mouse, the radiance measured in the intestines of infected, vehicle treated mice was above the background, whereas radiance measured in the intestines of infected mice treated with ciprofloxacin or ampicillin were all at approximately background levels. Bacteria counts quantified by plating intestine homogenates gave comparable results to the radiance values, although a significant difference (*P* < 0.0001) was observed between the ciprofloxacin and ampicillin treated groups (Fig. 6c). The plated tissue homogenates demonstrated mice treated with ciprofloxacin were essentially cleared of *S. flexneri* in the SI and cecum/LI 24 hours p.i. (Fig. 6c). Similar to ciprofloxacin, ampicillin generated a reduction in *Shigella* levels compared to vehicle control mice; however, the SI and cecum/LI of ampicillin treated mice had higher levels of *S. flexneri* compared to mice treated with ciprofloxacin (SI: *P* < 0.0001, cecum/LI: *P* < 0.0001) (Fig. 6c). Although the limit of detection is higher using the IVIS system, there was a positive linear correlation between the average radiance and bacterial counts in intestinal homogenates for both the SI (R^2^ = 0.63, *P* < 0.0001) and cecum/LI (R^2^ = 0.48, *P* < 0.01) (Fig. 6d).

### Ciprofloxacin pharmacokinetic/pharmacodynamic relationship with the mouse model of *S. flexneri* infection

**Figure 7:**
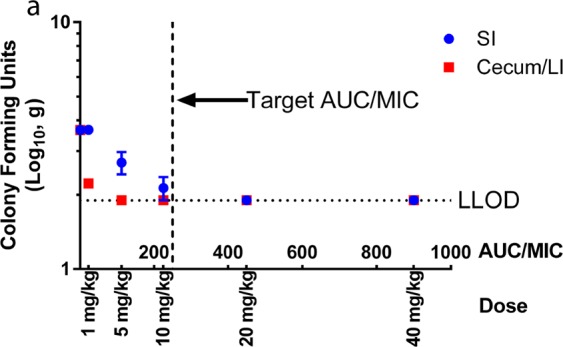
Ciprofloxacin dose response in the mouse model of *Shigella* infection. Previous studies have established a target ciprofloxacin AUC/MIC ratio of 250 for the treatment of Gram-negative bacterial infections in humans. The efficacy of ciprofloxacin oral doses predicted to generate AUC/MIC values ranging from 22–900 were evaluated in the mouse model of *Shigella* infection. In agreement with the target AUC/MIC ratio of 250, infected mice with predicted ciprofloxacin AUC/MIC values of ≤225 had detectable *S. flexneri* infection in the large intestine and/or small intestine. In contrast, mice receiving ciprofloxacin doses of 20 and 40 mg/kg BID (AUC/MIC ratios of 450 and 900, respectively) had no detectable *S. flexneri* in their gastrointestinal tissue. Values are reported as mean ± SD (n = 3 mice/group).

Previous studies have suggested a target AUC/MIC value of 250 for ciprofloxacin treatment of various Gram-negative bacterial infections in humans^24,25^. To determine if a similar pharmacokinetic/pharmacodynamic relationship was associated with ciprofloxacin efficacy against *S. flexneri* infection in mice, the efficacy of ciprofloxacin was evaluated with five doses ranging from 1–40 mg/kg BID. The ciprofloxacin doses were predicted to generate AUC/MIC values ranging from 20–900 based on the observed MIC of 0.008 µg/mL and previous experiments to characterize the intravenous and oral pharmacokinetics of ciprofloxacin in mice^26^. In agreement with the target AUC/MIC value of 250, mice with predicted AUC/MIC values of 450 and 900 (20 and 40 mg/kg BID ciprofloxacin) had no detectable *S. flexneri* in the small intestine and large intestine (Fig. 7). However, mice dosed with 1, 5, and 10 mg/kg ciprofloxacin BID had *S. flexneri* in the cecum/large intestine and/or small intestine. While the infection levels decreased in a dose dependent manner, the predicted AUC/MIC values were less than 250 for the 1, 5, and 10 mg/kg dose groups.

## Discussion

As shigellosis is one of the leading causes of diarrheal death in children under five and a major burden in resource limited countries^1,2^, it is critical to develop tools to investigate novel treatment options. Robust *in vitro* and *in vivo* models are required to transition drug candidates from the pre-clinical to clinical stages of development. A major hurdle in the development of *Shigella* models has been the lack of a *S. flexneri* strain that expresses a sensitive reporter which can be used to characterize *Shigella* growth and invasion. This work describes the development of a bioluminescent *Shigella* strain. Advantages of the luciferase system include: a lack of photo bleaching^27–29^, low background signal^30^, and a shorter half-life compared to GFP^31,32^. While the luminescence signal declines within hours of reaching a stationary phase of growth, the strain was suitable for the mammalian cell co-culture assays and *in vivo* assays. Further, there were no observed changes in MIC values between *S. flexneri* lux1 and the parent strain M90T Sm *S. flexneri*, suggesting the insertion of *luxCDABE* did not alter antibiotic susceptibility to the drugs tested.

While MIC values provide an initial assessment of antibiotic efficacy, translation of the MIC to *in vivo* efficacy is unclear. The life cycle of *Shigella in vivo* includes gastrointestinal epithelial membrane invasion, suggesting antibiotics will require cellular penetration and action for *in vivo* efficacy. Due to the perceived importance of cellular penetration, it has been suggested that gentamicin’s poor efficacy against *Shigella* in the clinic is due to its low permeability^33^. Assays to characterize *Shigella* invasion *in vitro* have been described previously, and these assays have used gentamicin to remove extracellular bacteria based on the premise that gentamicin inefficiently penetrates the cell membrane^34,35^. However, recent studies have demonstrated several limitations with using gentamicin to specifically target extracellular bacteria^36–40^, and our work also suggests gentamicin may have a significant time dependent effect on intracellular *S. flexneri* concentrations in HCT-8 cells. However, the assay with HCT-8 cells is limited by an inability to distinguish between intracellular bacteria and bacteria attached to the extracellular surface. The effect of gentamicin on intracellular bacteria was seen at concentrations lower than those typically used in invasion assays. In addition, while brief 20–30 minute incubation times with gentamicin have been reported^41^, other researchers report incubation times of 1–2 hours^42–44^, which could potentially have an effect on intracellular bacteria. Based on these results and the interest in characterizing the efficacy of antibiotics, the previously published methods were modified to exclude gentamicin and allow for the assessment of intracellular and extracellular antibiotic efficacy *in vitro*.

When the efficacies of ciprofloxacin and ampicillin are characterized with an *in vitro* co-culture system, the antibiotics have distinct differences in their observed efficacy in this assay. While ciprofloxacin tends to have a larger effect on extracellular *S. flexneri*, ampicillin is more effective at decreasing intracellular bacteria compared to extracellular bacteria. While the reason for this difference is unclear at this time, the enhanced intracellular activity may be due to active uptake transport of ampicillin by PepT1 into the HCT-8 cells^45^. In addition, while ampicillin appears more efficacious than ciprofloxacin in this assay, at low concentrations ciprofloxacin has a larger effect on *Shigella* levels compared to ampicillin. The relative efficacy of ciprofloxacin and ampicillin at low concentrations in the HCT-8 assay reflects the observed MIC values for the two antibiotics. For our *in vitro* studies with *S. flexneri* and HCT-8 cells, the bacteria are spun down onto the HCT-8 cells followed by a 30-minute incubation to assist in *S. flexneri* invasion prior to antibiotic administration. However, it is possible that the observed antibiotic-dependent intracellular decline in *S. flexneri* may partially represent antibiotics acting on extracellular bacteria that die shortly after internalization.

When progressing pre-clinical candidates for treating *Shigella* infection, a major hurdle has been the lack of a mouse model for assessing antibiotic efficacy. Ideally, a mouse model to characterize therapeutic efficacy will include a route of infection that mimics the fecal-oral transmission in humans. However, adult wild-type mice are resistant to oral infection unless they are given an antibiotic cocktail prior to inoculation with *S. flexneri*^46^. In addition, mice aged ≤4 days have shown susceptibility to infection with oral administration of *S. flexneri*^47^ or 7 day old mice with disrupted Paneth cells^48^. Based on previous work demonstrating the lethal dose for intranasally administered *S. flexneri* is approximately 5 orders of magnitude lower for IFN-γ knockout mice compared to wild-type mice^49^, IFN-γ KO mice were orally administered *S. flexneri* to determine if the mice were susceptible to infection. However, based on the small weight loss and no observed difference in gastrointestinal radiance or *S. flexneri* CFUs compared to control mice, the IFN-γ KO mouse model was not used for further studies. Recently, it was shown that IP inoculation with *S. flexneri* can generate the symptoms of shigellosis in mice^18,23^. The infection was well characterized by the researchers, and the mice had increased expression of proinflammatory cytokines in the large intestine, excess secretion of mucin in the colon, and altered fecal pathology^18^. Although the model of shigellosis by IP *S. flexneri* inoculation has been used to characterize the efficacy of a vaccine^18^, the model had never been used to assess the efficacy of antibiotics. Therefore, the mouse model of shigellosis was adapted for characterizing the clinically relevant antibiotics ciprofloxacin and ampicillin.

In agreement with their use in the clinic, ciprofloxacin and ampicillin reduce the gastrointestinal levels of *Shigella* in a mouse model of shigellosis. While there was no significant difference in the average radiance observed in the gastrointestinal tissue of mice treated with ciprofloxacin and ampicillin, the plated tissue homogenate demonstrated ciprofloxacin had a greater effect on *Shigella* levels in the mouse model. In agreement with these findings, a previous randomized double-blinded clinical study comparing 5 days of treatment with ciprofloxacin (500 mg BID) and ampicillin (500 mg QID) suggested that ciprofloxacin may be superior to ampicillin for treating shigellosis based on bacteriologic failure and mean number of stools^50^. Furthermore, our data suggests the target AUC/MIC for the treatment of *Shigella* infection in mice is in agreement with a previously established AUC/MIC target for the treatment of Gram-negative infections with ciprofloxacin in humans^24,25^. The results of the mouse model are encouraging given that the route of infection and localization of the bacteria in the murine model differs from that in humans. An additional observation from the *in vivo* study was the radiance observed in the gastrointestinal tissue correlated with bacterial counts from the tissue homogenates. While future studies may require plating tissue homogenate to determine whether treated groups are significantly different, *S. flexneri* lux1 allows for the rapid preliminary assessment of antibiotic efficacy, greatly accelerating the rate at which new drugs can be screened *in vivo*. In addition, questions remain regarding the desired pharmacokinetic (PK)/pharmacodynamic (PD) properties for drugs used to treat enteric pathogens such as *Shigella*. These models will be able to assist in the identification of PK/PD indices that will be crucial for designing studies as drugs progress toward clinical use. Taken together, these *in vitro* and *in vivo* models with *S. flexneri* lux1 provide a unique, robust platform to rapidly screen and characterize potential anti-shigellosis therapeutics in a high throughput manner.

## Materials and Methods

### Bacterial strains

*S. flexneri* strain M90T Sm *S. flexneri* (ATCC BAA-2402) was purchased from the American Type Culture Collection (ATCC, Manassas, VA) and cultured according to the manufacturer with slight modifications. Broth cultures of *Shigella* were grown at 37 °C and shaken at 220 rpm. Trypticase soy broth (TSB) was used for the liquid culture of *S. flexneri* M90T Sm. For plate cultures of the bacteria, agar forms of each broth were used along with the addition of 0.01% Congo Red. 30 µg/mL streptomycin was used for selection of the M90T strain. For mammalian cell co-culture assays and murine infection assays, 0.1% sodium deoxycholate was added to the broths to promote expression of proteins involved in *Shigella* invasion^51^. For all assays, frozen stocks of bacteria were spread on agar plates and grown overnight. The agar plates were stored at 4 °C and used for up to two weeks to start overnight broth cultures.

### Insertion of *luxCDABE* gene into M90T Sm chromosome

To generate a strain of *S. flexneri* that contains the *luxCDABE* cassette, a previously published protocol was used with slight modifications^19^. Plasmid pBEN276, a gift from Pierre Germon (Addgene plasmid # 69150) was designed for integration of the luciferase operon in the Tn7 insertion site of Gram-negative bacteria. Plasmid pBEN276 was purified from *E. coli* using a ZymoPURE Plasmid Midiprep Kit (Zymo Research, Irvine, CA) according to the manufacturer. The purified plasmid was concentrated using ethanol precipitation. M90T Sm *S. flexneri* was grown to log phase (3 hours) and centrifuged at 2,000 × *g* for 10 minutes. The resulting pellet was washed 3x with ice cold DNase/RNase-free distilled water (Thermo Fisher Scientific, Waltham, MA), and resuspended in 2.5 mL of water. 19 µL of the resuspended bacteria pellet was placed in each well of an Amaxa 6 × 16 Nucleocuvette™ Plate (Lonza, Allendale, NJ). A 96-well Shuttle™ device was used with the Amaxa 4D-Nucleofector™ System for optimization of Nucleofection™ conditions. Multiple conditions were tested, and GB 200 was chosen for the final electroporation setting. Once the electroporation program was complete, the contents of each well were placed in 100 µL S.O.C. media (Thermo Fisher Scientific, Waltham, MA) and incubated at 37 °C for 1 hour. Next, the 100 µL was added to 900 µL of lysogeny broth (LB) with a final ampicillin concentration of 100 µg/mL and shaken at 220 rpm for 16 hours. The O.D. of the overnight culture was determined for each well, and wells with an O.D. value greater than background were streaked on LB agar plates with 100 µg/mL ampicillin. After 16 hours at 37 °C, 5–10 colonies were selected from the plate and placed in TSB with arabinose to induce transposition. Following growth for 16 hours, the bacteria was streaked on TSB agar plates and grown at 42 °C for 16 hours to cure the plasmid. Individual colonies were selected and grown in TSB with 30 µg/mL streptomycin at 37 °C overnight. The following day, each culture was diluted 1:100 to inoculate a 2 mL culture. After 3 hours, the luminescence of each culture was measured with an EnVision Plate Reader (Perkin Elmer, Waltham, MA). The relative luminescence units (RLU) of each sample was used to identify the clone with the largest signal. This clone was flash frozen with liquid nitrogen in broth containing 10% glycerol and was used for all further studies. In addition, the clone was grown in 100 µg/mL ampicillin to ensure that the plasmid was cured and separately with 30 µg/mL streptomycin to demonstrate the clone retained resistance to streptomycin.

### Characterization of luciferase-expressing *S. flexneri*

To characterize the *in vitro* growth rate of *S. flexneri* lux1, an overnight *S. flexneri* lux1 broth culture was diluted both to a nominal OD600 of 0.05 and 1:100 in a white polystyrene, flat bottom 96 well plate (Corning, Corning, New York). With the *S. flexneri* lux1 strain and the reported culture conditions, overnight cultures consistently have an OD600 of ~0.8. The dilutions were performed in triplicate for each (OD600 = 0.05 and 1:100). Each culture was grown at 37 °C and shaken at 220 rpm. At set time points from 0.25–12 hours, four aliquots were removed from each incubation (n = 24/timepoint) with OD600 measured with two aliquots and luminescence measured with the remaining two. The average of the two duplicates was calculated for both the OD600 and the luminescence. The average and standard deviation of the three incubations for each time point were calculated and plotted as a function of time. To determine if there was a significant association between the luminescence and the OD600, the luminescence was plotted against its corresponding OD600 value. To determine the limit of detection for the bioluminescent *S. flexneri* lux1 and if there was an association between the luminescence and *S. flexneri* CFU, an overnight broth culture of *S. flexneri* lux1 was diluted 1:100. After 4 hours of growth, the cultures were serially diluted in triplicate and plated in a white 96 well plate. The same culture was plated on agar plates to determine the CFU present in the culture. In addition, the association between the luminescence and CFU was characterized for *S. flexneri* lux1 with the IVIS Spectrum.

### MIC characterization

To initially assess the *in vitro* potency of antibiotics with each *S. flexneri* strain, an established microdilution method was used to measure the MIC^52^. Antibiotics were plated in serial 2-fold dilutions with three replicates for each concentration. An overnight broth culture was used to add 5 × 10^5^ CFU/mL bacteria to each well. AirPore tape sheets (Qiagen, Inc., Venlo, Netherlands) were placed on each plate, and plates incubated at 37 °C for 22–24 hours with shaking at 220 rpm. The MIC was determined by reading absorption at 600 nm using an ELx800 Absorbance Microplate Reader (BioTek). The MIC was considered the lowest concentration in which absorbance decreased ≥90% compared to no drug controls. Assays were performed in duplicate on separate days to confirm MIC values.

### Mammalian cell co-culture assay to assess antibiotic efficacy

Human ileocecal adenocarcinoma (HCT-8) cells (ATCC) were plated in 96-well tissue culture treated plates (Thermo Fisher Scientific) grown in Roswell Park Memorial Institute 1640 (RPMI-1640) medium supplemented with 10% fetal bovine serum and 1% penicillin/streptomycin. 25,000 cells/well were grown to confluency at 37 °C in a 5% CO_2_ humidified incubator in 100 µL of media. One day prior to bacterial inoculation, the cells were washed with Dulbecco’s phosphate-buffered saline (DPBS), and fresh antibiotic-free RPMI-1640 medium was added to each well. On the day of inoculation, an overnight broth culture of *S. flexneri* lux1 was diluted 1:100 and grown to log-phase (4 hours). The luminescence of the log-phase bacteria was measured and inoculated into the well plate at an estimated multiplicity of infection (MOI) of 100.

The 96-well plates were spun at 2000 × *g* for 10 minutes to aid bacterial invasion and incubated for 30 min at 37 °C in a 5% CO_2_ humidified incubator. For studies using antibiotics, the antibiotics were added in serial 2-fold dilutions with 8 replicates. Following addition of antibiotics, the plates were incubated an additional 30 minutes to 4 hours. Following incubation, 80 µL of media was moved into a white polystyrene, flat bottom 96-well plate (Corning Inc., Corning, NY). The remaining 20 µL of media was removed and the HCT-8 cells were washed 3x with DPBS and lysed with 100 µL 0.1% Triton-X 100, shaking at 90 rpm at 37 °C for 5 minutes. 80 µL of cell lysate was moved into a separate white polystyrene, flat bottom 96-well plate. Bioluminescence of the media and cell lysate were determined on an EnVision Plate Reader (Perkin Elmer).

### Development of *in vivo* mouse model of shigellosis

An overnight *S. flexneri* lux1 broth culture was diluted 1:100 and grown to log-phase (3–4 hours). Log-phase bacteria were then centrifuged for 10 minutes at 2000 × *g*, and the resulting pellet was washed in DPBS without calcium and magnesium (Thermo Fisher Scientific) and centrifuged again for 10 minutes at 2000 × *g*. The pellet was resuspended in DPBS to a final concentration based on the target dose of *S. flexneri*. Each dose was confirmed using an EnVision Plate Reader (Perkin Elmer).

For testing a potential oral infection model in interferon gamma knockout (IFN-γ KO) mice, female IFN-γ KO mice (002287, Jackson Laboratories, Bar Harbor, ME) aged 9–10 weeks were weighed and infected via oral gavage (PO) with escalating infections of *S. flexneri* lux1 or PBS vehicle control (n = 3 per group). The escalating doses were 5 × 10^8^, 1 × 10^9^, or 5 × 10^9^ CFU/mouse. Mice were subsequently weighed at 2, 6, and 24 hours post infection (p.i.). Mice were euthanized 24 hours p.i. and the whole small intestine and whole cecum and large intestine were collected.

To perform the dose escalation experiment with luciferase-expressing *S. flexneri* lux1, female B6 mice (000664 Black 6, Jackson Laboratories) aged 9–10 weeks were weighed and infected IP with escalating infections of *S. flexneri* lux1 or vehicle control (n = 3 per group). The escalating bacterial doses were 5 × 10^7^, 5 × 10^8^, 1 × 10^9^, or 5 × 10^9^ CFU/mouse. Mice were weighed, and their organs were collected as described above.

To determine the background radiance of gastrointestinal tissue from mice on the IVIS system, female B6 mice (000664 Black 6, Jackson Laboratories) aged 9–10 weeks were treated with vehicle control, euthanized and organs were collected as described above.

### Characterization of ampicillin and ciprofloxacin efficacy in the mouse model

An overnight *S. flexneri* lux1 culture was prepared as described above and resuspended in DPBS to a final concentration based on the target dose of 5 × 10^7^ CFU/mouse. Each dose was confirmed using an EnVision Plate Reader (Perkin Elmer). Female B6 mice (000664 Black 6, Jackson Laboratories) aged 9–10 weeks were weighed and infected IP with 5 × 10^7^ CFU/mouse (total n = 10 per group, experiments performed in duplicate on separate days, with n = 5 per group for each duplicate). Mice were subsequently weighed at 2, 16, and 24 hours p.i. Antibiotics were suspended in oral vehicle (3% ethanol/7%Tween 80/90% saline), and mice were administered antibiotics or vehicle control PO at 2 and 16 hours p.i. Ciprofloxacin was administered at 40 mg/kg and ampicillin at 50 mg/kg of body weight. Mice were euthanized 24 hours p.i. and the whole small intestine and whole cecum and large intestine were collected.

To determine if there was an association between the ciprofloxacin exposure and *in vivo* efficacy, five dosing regimens were evaluated in the mouse model of *Shigella* infection. It has been previously reported that a AUC/MIC of 250 is associated with ciprofloxacin efficacy against Gram-negative bacterial infections in humans^24,25^. With a ciprofloxacin MIC value of 0.008 µg/mL against *S. flexneri* lux1 (Table 1), previously published pharmacokinetic studies in mice were used to select doses that would generate AUC/MIC values ranging from 22–900^26^. The reported intravenous and oral ciprofloxacin pharmacokinetics in mice suggested there will be no ciprofloxacin remaining 12 hours after the first oral ciprofloxacin dose in mice, so accumulation of drug was not expected with BID dosing over a 24-hour study. A ciprofloxacin dose of 50 mg/kg generated an AUC_0-inf_ of 4.5 µg*hr/mL, and this AUC was used to predict the AUC_0–24hr_ for each ciprofloxacin dose in the PK/PD efficacy studies^26^. When administered orally BID, the predicted AUC values for 1, 5, 10, 20, and 40 mg/kg ciprofloxacin were 0.18, 0.9, 1.8, 3.6, and 7.2 µg*hr/mL, respectively. The AUC values and the ciprofloxacin MIC value of 0.008 µg/mL were used to calculate the AUC/MIC values 22, 113, 225, 450, and 900. The efficacy experiment was performed as described above (n = 3 mice/group). The mouse tissues were harvested at the end of the experiment as described for tissue homogenate plating.

### Detection and quantification of *S. flexneri* lux1 with IVIS and tissue homogenate plating

Organs were imaged using an IVIS Spectrum (Perkin Elmer) to determine the levels of *S. flexneri* infection. For the analysis, Living Image (Perkin Elmer) was used to quantify the average radiance for *S. flexneri* lux1. A region of interest (ROI) was drawn separately to encompass the small intestine or the cecum/large intestine. The ROIs for the small intestine and large intestine were drawn in a way that ensured all gastrointestinal tissue (small intestine or large intestine) for every mouse would be contained within the ROI without interference from the other intestinal section. This ROI was used to analyze each sample from every mouse in the study. For the initial antibiotic efficacy experiments with 50 mg/kg ampicillin and 40 mg/kg ciprofloxacin BID, the observed radiance values were normalized by the weight of the tissue and to background signal based on the average radiance of uninfected mouse intestines. Gastrointestinal tissue was also resuspended in 1 g/mL PBS, homogenized, and plated on TSB agar plates with 30 µg/mL streptomycin to confirm infection levels.

### Animal ethics statement

All animal procedures and protocols were approved by the institutional animal care and use (IACUC) committee at the University of Washington (protocol Number: 2154-01) and all efforts were made to minimize animal discomfort and suffering. The University of Washington is accredited by the Association for the Assessment and Accreditation of Laboratory Animal Care, International (AAALAC). The Office of Laboratory Animal Welfare of the National Institutes of Health (NIH) has approved the University of Washington (#A3464-01), and this study was carried out in strict compliance with the Public Health Service (PHS) Policy on Humane Care and Use of Laboratory Animals.

### Statistics

GraphPad Prism software (GraphPad, La Jolla, CA) was used for statistical analysis. For determining linear relationships, linear regression analysis was performed. For *in vivo* experiments, bacterial counts/radiance in the intestines and mouse weights were compared using ANOVA and unpaired *t*-tests. Data were considered statistically significant for *P* ≤ 0.05.

## Supplementary information

Supplemental Figure 1

## Data Availability

The datasets generated during and/or analyzed during the current study are available from the corresponding author on reasonable request.
