# Author Correction: Evaluation of *in vitro* and *in vivo* antibiotic efficacy against a novel bioluminescent *Shigella flexneri*

**DOI:** 10.1038/s41598-020-59766-x

**Published:** 2020-02-11

**Authors:** Molly C. McCloskey, Shareef Shaheen, Lesley Rabago, Matthew A. Hulverson, Ryan Choi, Lynn K. Barrett, Samuel L. M. Arnold

**Affiliations:** Department of Medicine, Division of Allergy and Infectious Diseases, and the Center for Emerging and Re-emerging Infectious Diseases (CERID), University of Washington, Seattle, WA 98109 United States

Correction to: *Scientific Reports* 10.1038/s41598-019-49729-2, published online 19 September 2019

The Acknowledgements section in this Article is incomplete.

“The authors recognize Wesley C. Van Voorhis MD, PhD of the University of Washington for his thoughtful discussions on the development of the models.”

should read:

“The authors recognize Wesley C. Van Voorhis MD, PhD of the University of Washington for his thoughtful discussions on the development of the models. This work was funded by Tres Cantos Open Lab Foundation (Grant: TC246).”

