# Supplementary material for: Evaluation of in vitro and in vivo antibiotic efficacy against a novel bioluminescent Shigella flexneri
Source: Sci Rep. 2019 Sep 19;9:13567. doi: 10.1038/s41598-019-49729-2 (PMC6753072; doi:10.1038/s41598-019-49729-2)
Supplement: Supplementary file 1 — Supplemental Figure 1 [file 41598_2019_49729_MOESM1_ESM.docx]

Evaluation of in vitro and in vivo antibiotic efficacy against a novel bioluminescent *Shigella flexneri*

Molly C. McCloskey^a^, Shareef Shaheen^a^, Lesley Rabago^a^, Matthew A. Hulverson^a^, Ryan Choi^a^, Lynn K. Barrett^a^, Samuel L. M. Arnold^a*^

^a^Department of Medicine, Division of Allergy and Infectious Diseases, and the Center for Emerging and Re-emerging Infectious Diseases (CERID), University of Washington, Seattle, WA 98109, United States

*Corresponding author

**Supplementary Data Figure Legends and Tables**

**
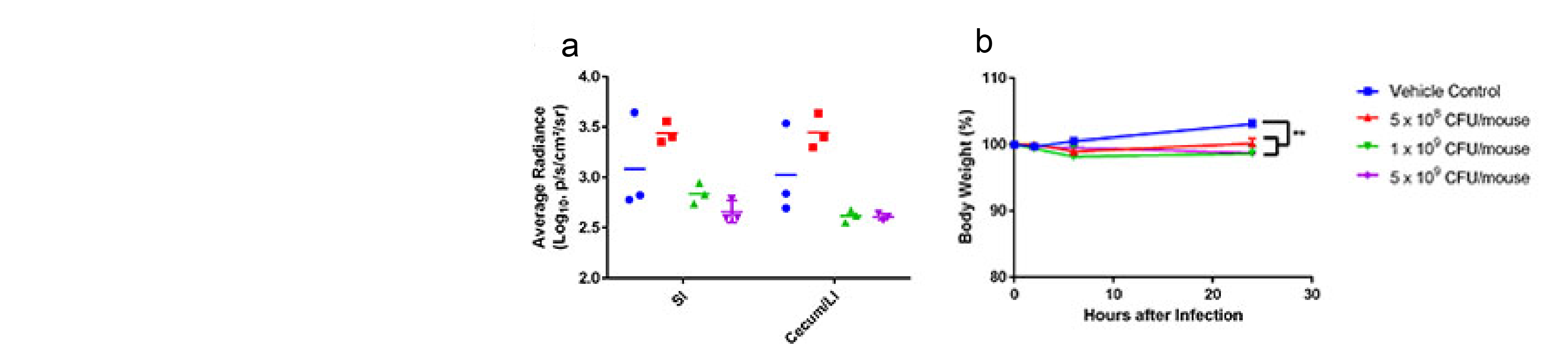
**

Supplemental Fig. 1: Oral inoculation of adult IFN-γ KO mice with *S. flexneri* lux1. 24 hours after IFN-γ KO mice were orally administered doses of *S. flexneri* lux1 (5 x 10^8^, 1 x 10^9^, or 5 x 10^9^ CFU/mouse), the average radiance was measured in the SI and cecum/LI using IVIS (a). In addition, the body weight of IFN-γ KO mice infected with escalating doses of *S. flexneri* lux1 or vehicle control were compared (b). There was a small but significant (*P* < 0.01) difference in the body weight of infected mice compared to vehicle controls. For the control and infected mice, the weights and tissue radiance were compared with a Student’s two tailed t-test. n=3 mice/group. **- P* < 0.05, **- *P* < 0.01, ***- *P* < 0.001.
